# A Comparative Study of the Rapid (I_Kr_) and Slow (I_Ks_) Delayed Rectifier Potassium Currents in Undiseased Human, Dog, Rabbit, and Guinea Pig Cardiac Ventricular Preparations

**DOI:** 10.3390/ph17081091

**Published:** 2024-08-20

**Authors:** Márta Ágoston, Zsófia Kohajda, László Virág, Beáta Baláti, Norbert Nagy, Csaba Lengyel, Miklós Bitay, Gábor Bogáts, András Vereckei, Julius Gy. Papp, András Varró, Norbert Jost

**Affiliations:** 1 Department of Pharmacology and Pharmacotherapy, Albert Szent-Györgyi Medical School, University of Szeged, P.O. Box 427, 6701 Szeged, Hungary; 2 HUN-REN-SZTE Research Group for Cardiovascular Pharmacology, 6701 Szeged, Hungary; 3 Interdisciplinary Research and Development and Innovation Centre of Excellence, University of Szeged, 6720 Szeged, Hungary; 4 Department of Medicine, Albert Szent-Györgyi Medical School, University of Szeged, 6720 Szeged, Hungary; 5 Department of Cardiac Surgery, Albert Szent-Györgyi Medical School, University of Szeged, 6742 Szeged, Hungary; 6 Department of Internal Medicine and Haematology, Semmelweis University, 1088 Budapest, Hungary

**Keywords:** antiarrhythmic drugs, K^+^ channel blockers, proarrhythmic action, cardiac action potential, ion currents

## Abstract

To understand the large inter-species variations in drug effects on repolarization, the properties of the rapid (I_Kr_) and the slow (I_Ks_) components of the delayed rectifier potassium currents were compared in myocytes isolated from undiseased human donor (HM), dog (DM), rabbit (RM) and guinea pig (GM) ventricles by applying the patch clamp and conventional microelectrode techniques at 37 °C. The amplitude of the E-4031-sensitive I_Kr_ tail current measured at −40 mV after a 1 s long test pulse of 20 mV, which was very similar in HM and DM but significant larger in RM and GM. The L-735,821-sensitive I_Ks_ tail current was considerably larger in GM than in RM. In HM, the I_Ks_ tail was even smaller than in DM. At 30 mV, the I_Kr_ component was activated extremely rapidly and monoexponentially in each studied species. The deactivation of the I_Kr_ component in HM, DM, and RM measured at −40 mV. After a 30 mV pulse, it was slow and biexponential, while in GM, the I_Kr_ tail current was best fitted triexponentially. At 30 mV, the I_Ks_ component activated slowly and had an apparent monoxponential time course in HM, DM, and RM. In contrast, in GM, the activation was clearly biexponential. In HM, DM, and RM, I_Ks_ component deactivation measured at −40 mV was fast and monoexponential, while in GM, in addition to the fast component, another slower component was also revealed. These results suggest that the I_K_ in HM resembles that measured in DM and RM and considerably differs from that observed in GM. These findings suggest that the dog and rabbit are more appropriate species than the guinea pig for preclinical evaluation of new potential drugs expected to affect cardiac repolarization.

## 1. Introduction

One possible way to treat cardiac arrhythmias is to prolong the duration of the ventricular action potential (APD) or effective refractory period (ERP) using Class III antiarrhythmic drugs [1,2]. The development of new antiarrhythmic drugs acting on the ion channels responsible for myocardial membrane repolarization has been the subject of intense research for two decades. The late rectifier potassium current (IK) is one of the transmembrane ionic currents, considered to be the most important regulator of ventricular repolarization [3,4]. Most antiarrhythmic agents that act by prolonging cardiac repolarization (class I/A and III) are inhibitors of this ionic current. This current was first described by Noble and Tsien in goat Purkinje fibers [5], but it has since been shown to be present in many other species and myocardial types [3,6,7,8]. The late rectifier K^+^ current (I_K_) is composed of a fast (I_Kr_) and a slow (I_Ks_) component in most species [7,9,10,11], including humans [12]. The two components differ in both drug sensitivity and the nature or kinetics of their voltage–current dependence [6,7,10,13].

Prior to this series of studies, both current components were thought to play a significant role in normal ventricular action potential repolarization [8,10,11,14]. Selective I_Kr_ blockers [e.g., d-sotalol (Bristol Myers, Evansvile, IN, USA), E-4031 (Merck-Sharp&Dohme Research Laboratories, Rahway, NJ, USA), dofetilide (Merck-Sharp&Dohme Research Laboratories, Rahway, NJ, USA) significantly prolong myocardial cell APD [1,15,16], consistent with the strong antiarrhythmic effects of some of their representatives demonstrated in humans [2,17]. All I_Kr_ blockers show a reverse use-dependence [18], i.e., they provide more potent repolarization at low heart rates than at higher frequencies. The antiarrhythmic effects of these agents during repolarization are therefore the least pronounced at abnormally high heart rates, whereas their APD-enhancing effects are most pronounced in bradycardia. After a long diastolic interval or at slow heart rate, I_Kr_ blockers significantly prolong APD, which favors early afterdepolarization and the consequent *Torsades de Pointes*-type ventricular tachycardia (TdP) [17]. Based on their experiments on guinea pig cardiac myocytes, Jurkiewicz and Sanguinetti hypothesized that selective inhibition of I_Ks_ channels increases the frequency, effective refractory period (ERP) and APD in a heart rate-independent manner [15]. The development of selective IKs blockers was greatly stimulated by the expectation that a new class of agents that conformed to the above hypothesis would have antiarrhythmic effects that would provide repolarization without the risk of TdP. However, since selective IKs blockers only became available in the early 2000s [10,19,20], the physiological and pharmacological effects of IKs currents on cardiac repolarization have been investigated in several mammalian species, including humans, for the first time.

The I_Kr_ and I_Ks_ data from the different species reported in the literature are highly variable and often contradictory [6,10,11,12,13,21]. These differences may be explained by the different experimental methods used. There may be differences in both the cell isolation and assay techniques used for the measurements. Moreover, the latter may also depend to a large extent on the knowledge available at the time the test was performed. This naturally raises the question of how these animal data can be applied to humans. Since it is very difficult to obtain human myocardial tissue for experimental purposes, especially from healthy donors, the properties of these two important currents in healthy humans have not been known until now. Also, the limited data that are known so far come only from myocardial cells from diseased explanted hearts [12,21,22]. In addition, the two agents used in this study to inhibit calcium and inward rectifying potassium currents, CdCl_2_ [12,21] and BaCl_2_ [12], are known to alter the kinetic properties of I_Kr_ and I_Ks_ currents [23,24]. Therefore, these data can only be interpreted with caution. 

When designing new antiarrhythmic drugs, significant preclinical studies precede clinical trials of new agents. These studies are performed in various animal models, and the resulting data are used to decide whether an agent can be tested in human clinico-pharmacological studies. However, on the one hand, the I_Kr_ and I_Ks_ currents in the human heart are currently unknown, and on the other hand, we have seen that the various animal data available are often contradictory. It would therefore be important to know which animal species are the most “human”, at least in terms of I_Kr_ and I_Ks_ currents, which are so important from the point of view of antiarrhythmic agents. The aim of the present study is to fill these knowledge gaps. The only way to answer this question is to test the two currents under similar experimental conditions (both cell isolation techniques and experimental assay protocols) using healthy human cardiac myocytes and a number of other species that are currently used for preclinical studies.

This was the first series of studies used to investigate the properties of I_Kr_ and I_Ks_ currents in ventricular preparations from human donor hearts (used for heart valve transplantation) using conventional and patch clamp techniques. In parallel to these experiments, our research group investigated the properties of I_Kr_ and I_Ks_ currents in ventricular preparations from canine, rabbit, and guinea pig hearts under similar experimental conditions. These are currently the most frequently used species for in vitro and in vivo preclinical testing of antiarrhythmic agents, as they are the species in which the presence of both currents has been previously clearly demonstrated [7,10,11]. In this study, we were able to determine which of these species is most similar to humans, and we also identified and reported significant new knowledge about the physiological role of I_Kr_ and I_Ks_ currents in ventricular repolarization.

## 2. Results

### 2.1. Ion Current Measurements Using the Patch Clamp Technique

Experiments were performed on ventricular myocardial cells from canine, rabbit, guinea pig, and healthy human donor hearts.

#### I_Kr_ and I_Ks_ Currents in Undiseased Human, Canine, Rabbit, and Guinea Pig Cardiomyocytes

**Figure 1:**
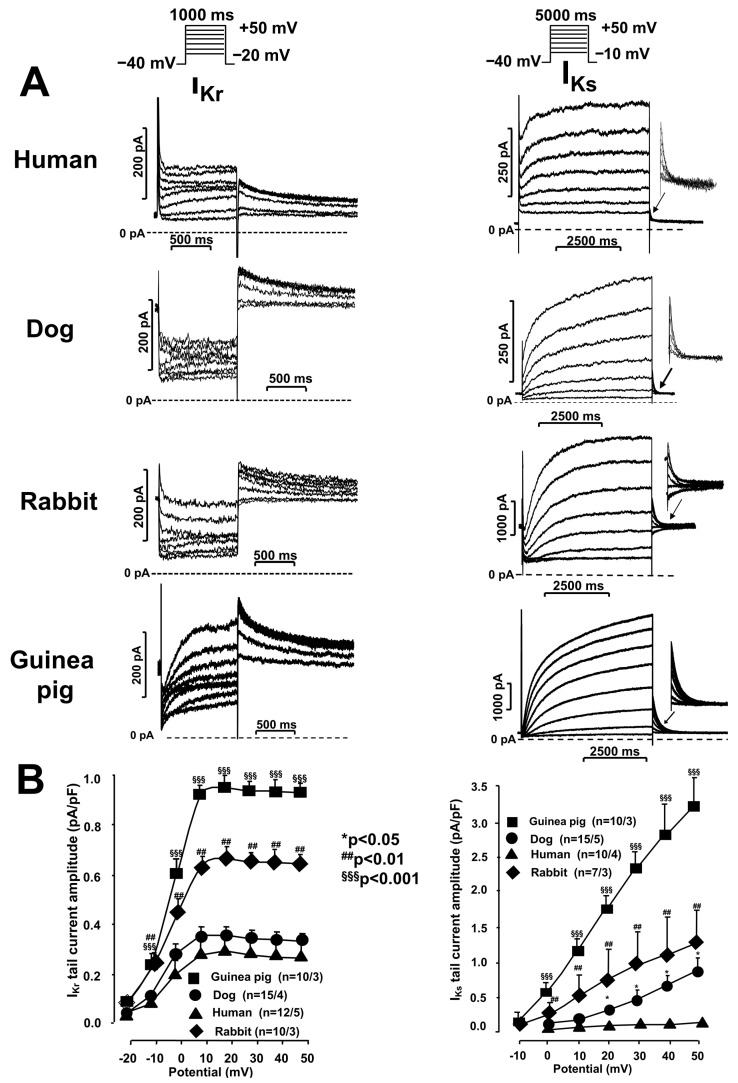
(**A**) Original recordings of the rapid component (I_Kr_, **left** panels) and slow component (I_Ks_, **right** panels) of the delayed rectifier potassium current measured in undiseased human, dog, rabbit, and guinea pig ventricular myocytes. To determine the I_Kr_ and I_Ks_ currents, the cells were depolarized with 1000 ms (for I_Kr_) or 5000 ms (for I_Ks_) long test pulses, respectively, between −20 mV and 50 mV, from a holding potential of −40 mV. The deactivating current (“tail-current”) observed after repolarization to −40 mV was considered as the I_Kr_ or I_Ks_ current. The pulse frequency was 0.05 Hz (for I_Kr_) or 0.1 Hz (for I_Ks_), respectively. The I_Kr_ current was measured in the presence of 100 nM L-735,821, while the I_Ks_ current was measured in the presence of 1–5 µM E-4031. A total of 1 µM nisoldipine was used to inhibit the L-type calcium current (I_CaL_). (**B**) Corresponding current–voltage (I-V) characteristics of I_Kr_ (**left** diagram) and I_Ks_ (right diagram) currents in isolated ventricular myocardial cells from human (triangle), dog (circle), rabbit (diamonds), and guinea pig (rectangle) heart preparations. The number of values (*n*) reflect the cell number/animal, given separately. * denotes significance between human and dog; ^##^ denotes significance between human and rabbit; ^§§§^ denotes significance between human and guinea pig.

Figure 1 presents original I_Kr_ and I_Ks_ recordings (Figure 1A) and the corresponding current–voltage (I–V) characteristics (Figure 1B bottom panels) of the I_Kr_ and I_Ks_ currents. As can be seen in the left panels of Figure 1A,B, the I_Kr_ current, although with different amplitudes, is present in all the species studied. The amplitude of the E-4031-sensitive I_Kr_ tail current determined at a +20 mV membrane potential was very similar in human and canine cardiomyocytes (0.35 ± 0.07 pA/pF and 0.38 ± 0.02 pA/pF, *n* = 12–15, respectively) but higher in rabbit and guinea pig myocytes (0.66 ± 0.05 pA/pF and 1.0 ± 0.08 pA/pF, *n* = 10, respectively).

IKs currents (right panels of Figure 1A,B) were present in all four species studied, but their magnitude was again significantly species-dependent. The I_Ks_ tail current determined at a +50 mV membrane potential was significantly larger in the guinea pig (3.3 ± 0.6 pA/pF, *n* = 10) than in the rabbit (1.22 ± 0.7 pA/pF, *n* = 7) and dog ventricular myocytes (0.9 ± 0.05 pA/pF, *n* = 24). In the human ventricular myocytes, the I_Ks_ current was significantly lower, even compared to that measured in the dog cells (0. 2 ± 0.05 pA/pF, *n* = 14). Figure 1 shows that the I_Kr_ current starts to activate at more negative voltages (−20 mV) than the IKs component (–10 to 0 mV). The amplitude of the I_Kr_ current increases rapidly as the voltage increases and reaches its maximum at positive voltages, somewhere within the +10 to +20 mV membrane potential range. At voltages more positive than 20 mV, the I_Kr_ tail current does not increase any further. Moreover, an apparent decrease could be observed in all four investigated species (Figure 1, left diagram). This is called inward rectification. The I_Ks_ current did not appear to inwardly rectify (Figure 1, right diagram).

### 2.2. Activation and Deactivation Kinetics of I_Kr_ and I_Ks_ Currents 

**Figure 2:**
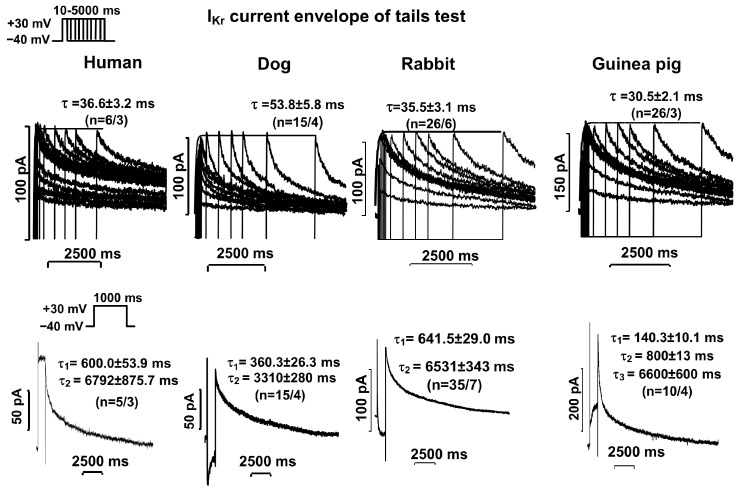
Kinetics of I_Kr_ current activation (**upper** fields) and deactivation (**lower** fields) in human, dog, rabbit and guinea pig isolated ventricular myocardial cells. The activation kinetics of I_Kr_ were studied by applying the envelope of tails protocols. Currents were elicited via depolarization from −40 mV to 30 mV using pulses ranging from 10 ms to 5000 ms in duration. Tail currents were recorded after repolarization to −40 mV with a pulse frequency of 0.05 Hz. At +30 mV, the I_Kr_-tail current presented fast and monoexponential activation kinetics in all the studied species. The deactivation kinetics of the I_Kr_ outward tail current were measured at −40 mV after a 1000 ms long test pulse to +30 mV. The deactivation was characterized by the time constant (*τ*) of the exponential function fitted to the deactivating tail current measured on the return to −40 mV. The corresponding activation and deactivation time constants (mean ± SEM) are given separately for each species.

**Figure 3:**
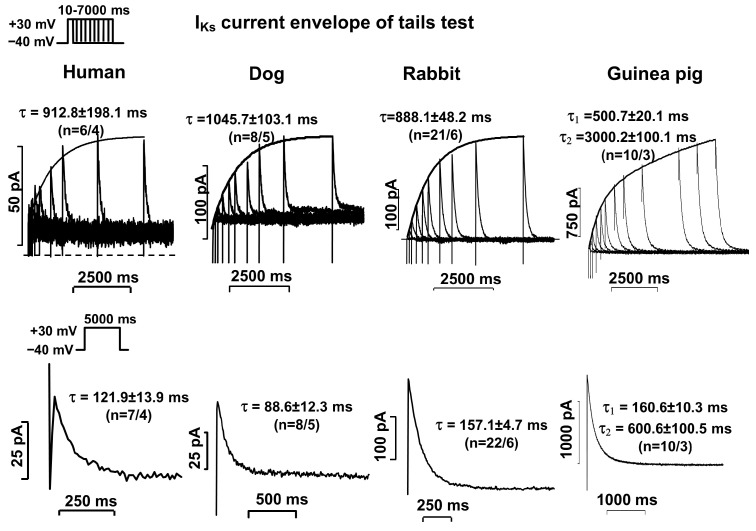
Activation (**upper** panels) and deactivation (**lower** panels) kinetics of I_Ks_ current in isolated human, canine, rabbit, and guinea pig ventricular myocardial cells. Similar envelope of tail test protocols were used. Activation kinetics of I_Ks_ were measured as tail currents at −40 mV, after test pulses of +30 mV, with durations gradually increasing from 10 to 5000 ms (or 7000 ms in the case of the guinea pigs). Deactivation kinetics of I_Ks_ outward tail current were measured at −40 mV after a 5000 ms test pulse to +30 mV with a pulse frequency of 0.1 Hz. The deactivation was characterized by the time constant (*τ*) of the exponential function fitted to the deactivating tail current measured on the return to −40 mV. The corresponding activation and deactivation time constants (mean ± SEM) are given separately for each species.

The activation of I_Kr_ and I_Ks_ currents was determined by measuring the current deactivating to −40 mV after test pulses that depolarized from −40 mV to +30 mV, with increasing durations from 10 ms to 5000–7000 ms (the so-called “envelope of tail test” protocol). The pulse frequency was 0.05 Hz for I_Kr_ current measurements or 0.1 Hz for I_Ks_ current measurements. The activation was expressed by the time constant of the exponential function fitted to the current amplitude–test pulse length relationship. Deactivation was expressed by the time constant of the exponential function (s) fitted to the tail current measured on the return of the test potential from +30 mV to −40 mV (see voltage protocol inset in Figure 2 and Figure 3.

The activation of the I_Kr_ current (measured at 30 mV) was fast and had exponential kinetics in all four species studied (Figure 2, top panels, Table 1). The deactivation of the I_Kr_ current (measured at −40 mV after a 1000 ms pulse depolarizing to 30 mV) was slow and double exponential in the human, dog, and rabbit ventricular myocytes, whereas in the guinea pigs, the deactivation of the I_Kr_ current was best fitted by three exponential functions (Figure 2, bottom panels, Table 1). 

The activation of the I_Ks_ current in human, canine, and rabbit myocytes (measured at 30 mV) was rapidly and apparently activated exponentially, while in guinea pig cells, the activation was clearly double exponential (Figure 3, top panels, Table 1). In human, canine, and rabbit cardiac myocytes, the deactivation kinetics of the I_Ks_ current (measured at −40 mV after a 5000 ms long depolarizing pulse to 30 mV) were fast and apparently exponential, while in the guinea pig myocytes, in addition to the fast component, a slow component was clearly identifiable (Figure 3, bottom panels, Table 1).

The time constants for the activation and deactivation kinetics of the I_Kr_ and I_Ks_ currents of the four investigated species are summarized in Table 1.

### 2.3. Action Potential Measurements Using Conventional Microelectrode Techniques

**Figure 4:**
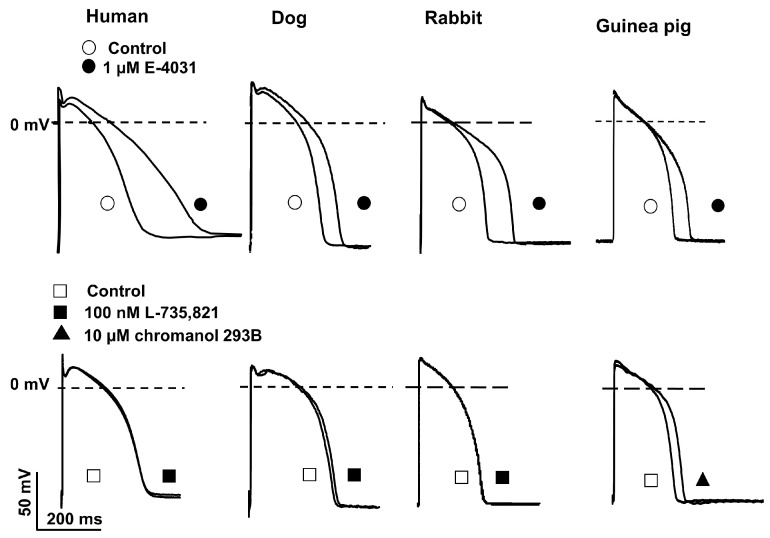
Representative transmembrane action potentials recorded from endocardial cells of the right ventricular papillary muscles of human, dog, rabbit, and guinea pig hearts after application of control (open circle) and I_Kr_ (1 µM E-4031, closed circle) (**upper** panels) and control (open rectangle) and I_Ks_ [(100 nM L-735,821 (closed rectangle) and 10 µM chromanol 293B (closed triangle)] (**lower** panels) blockers (after 40 min of exposure) at 1 Hz steady-state stimulation. The effects of chromanol 293B on the guinea pig action potential (lower right field) were investigated using 0.5 Hz stimulation. The recording presenting the effects of the I_Kr_ blocker E-4031 on the guinea pig action potential (upper right panel) was provided by the staff of the Department of Pharmacology and Toxicology, University of Dresden (see Acknowledgements). This study was performed under exactly the same experimental conditions (instrumentation, solutions, and experimental protocols) as those in our laboratory.

The effects of blocking I_Kr_ and I_Ks_ on the action potential were investigated using the conventional microelectrode technique. Representative action potential recordings show that I_Kr_ blocker E-4031 (1 µM) at a 1000 ms (1 Hz frequency) cycle length stimulation significantly (30–70%) elongated the API during 40 min incubation in all four species tested (Figure 4, upper panels). The I_Ks_ blocker L-735,821 (100 nM) at a 1000 ms cycle length stimulation did not cause significant changes in the human, dog, and rabbit papillary muscle APD (Figure 4, upper panels). In contrast, in the guinea pig right ventricular papillary muscle, the other selective I_Ks_ blocker, chromanol 293B (low concentration of 10 µM) [7], significantly elongated the APD by about 12% (Figure 4, lower right panel). In this experiment, the cycle length was 500 ms (0.5 Hz frequency) for more stable stimulation.

## 3. Discussion

### 3.1. Summary of Results of Previous Publications Describing I_Kr_ and I_Ks_ Current Measurements in Various Mammalian Species

It has long been known that in both Purkinje and ventricular myocardial fibers, there exists an outward current that is slowly activated and not inactivated during the plateau phase. This current has been suggested to be responsible for frequency-dependent changes in the action potential duration of Purkinje fibers [5]. Studies using the patch-clamp technique have demonstrated the presence of this current in a number of species, including humans [7,8,9,11,12,21,22,25], which has been termed the late rectifier potassium current (I_K_). A significant new finding by Sanguinetti et al. was the description of two components of the I_K_ current in experiments performed in guinea pig cardiac myocytes. They confirmed that there is a rapidly activating and deactivating component of the I_K_ current, named I_Kr_, and a slowly activating and deactivating component of the I_Ks_ current [11]. The two components differ in both drug sensitivity and the nature or kinetics of the voltage–current dependence [6,7,8,10,11]. It has long been known that the I_Kr_ current is blocked relatively selectively in the heart by several third-class antiarrhythmic agents, including d-sotalol, dofetilide, and the methanesulfonanilide derivative E-4031, which is close to the structure of dofetilide [6,11,17]. Jurkiewicz and Sanguinetti raise the possibility of a third-class antiarrhythmic agent with a new mechanism of action in its communication. Based on experimental results using guinea pigs, they raise the possibility that selective I_Ks_ blockers will prolong APD in a non-reversable frequency-dependent manner. This is inferred from the fact that, as a consequence of the fast I_Kr_ deactivation and slow I_Ks_ deactivation observed in guinea pigs, I_Ks_ current accumulates at shorter diastolic intervals. Thus, during tachycardia, selectively blocking the I_Ks_ component may lengthen the APD better than in bradycardia; i.e., it does not show a reverse use-dependence effect (“Sanguinetti hypothesis”) [15]. The development of selective I_Ks_ blockers was greatly stimulated by this hypothesis, i.e., it was supposed that selective I_Ks_ blockers (as a new class of antiarrhythmic agents) would provide repolarization lengthening (Class III) antiarrhythmic effects free from the risk of TdP. First, the I_Ks_-blocking effects of propofol and thiopentone were demonstrated in isolated guinea pig ventricular myocytes. However, in this study, Heath and Terrar used a different experimental approach [13]. The authors inhibited the very large I_Ks_ current present in guinea pigs with this new, selective I_Ks_ blocker thiopentone and were thus able to determine the kinetic properties of the residual I_Kr_ current more precisely. The experiments showed that the I_Kr_ channel activates rapidly but deactivates slowly and double-exponentially, while the I_Ks_ component activates slowly and deactivates rapidly. These results strongly question the validity of the Sanguinetti hypothesis [13,15].

The reasons for the two different results can be found in the very different experimental set-ups. In their experiments, Sanguinetti et al. used only a very short repolarizing pulse of 750 ms to study the deactivation kinetics and thus could easily overlook the second, slower component of the I_Kr_ current, which deactivates bilaterally [11,15]. On the other hand, the experiment performed by Heath and Terrar’s group in the presence of a I_Ks_ blocker is a more precise approach. Therefore, they could see and study the properties of the native I_Kr_ current more clearly [13]. In contrast, Sanguinetti et al. measured the I_Kr_ current only as the E-4031-sensitive difference current, which can be a significant source of error, especially when determining the kinetic parameters (e.g., fits with exponential functions) [11,15].

Effective I_Ks_-selective blockers appeared only in the late 1990s. These were the L-735,821 and L-768,673 compounds (synthetized by Merck-Sharp&Dohme Research Laboratories, Rahway, NJ, USA), respectively, and two chromanol derivatives, chromanol 293B and HMR-1556 (Hoechst-Marion-Roussel, Frankfurt, Germany, 20). Chromanol 293B was later found to have very poor selectivity and to block many other currents at the concentrations where I_Ks_ currents are also present [26]. This is important to note, because there have been a number of publications on the role of the I_Ks_ component in which compound 293B was used at very high concentrations. Additionally, the obtained results have been used to demonstrate the efficacy of I_Ks_ blockers as useful antiarrhythmic agents. This has been the subject of considerable debate in the literature [27,28].

The rabbit is a widely used animal model for testing antiarrhythmic agents both in vivo and in vitro. It was therefore important to establish the properties of this important repolarizing current in this species. An early study first detected only the rapidly activating (so-called E-4031-sensitive component) I_Kr_ component in rabbits, whereas the slowly activating E-4031-insensitive I_Ks_ component could not be identified [6]. Therefore, it was long assumed that I_Ks_ components were not present in rabbits. Later, however, Salata et al. succeeded in showing that I_Ks_ currents are present in rabbits, and with a very high amplitude, precisely by using the selective I_Ks_ blocking compound L-735,821 [10]. In the study of Salata et al., the reported I_Kr_ and I_Ks_ kinetics differed significantly from the guinea pig I_Kr_ and I_Ks_ values previously reported by Sanguinetti et al. In this study, the I_Kr_ component activated rapidly (≈30 ms) and deactivated slowly in a double-exponential manner (τ_1_ ≈ 300 ms and τ_2_ ≈ 3000 ms), while the I_Ks_ component activated slowly (τ ≈ 700 ms) and deactivated rapidly with exponential kinetics (τ ≈ 200 ms) [10].

Both current components have been detected in dogs by two working groups. Their results suggest that in dogs, I_Kr_ currents activate rapidly and deactivate slowly in a double-exponential manner (τ_1_ ≈ 200 ms and τ_2_ ≈ 2800 ms), whereas I_Ks_ currents activate slowly (τ ≈ 800 ms) and deactivate rapidly in an exponential manner (τ ≈ 150 ms) [7,8]. In other words, I_Kr_ and I_Ks_ currents in rabbits and dogs have very similar kinetic properties [7,8]. Based in these kinetic parameters, Gintant was the first to question the reliability of the Sanguinetti hypothesis [15] in 1996. He reported that it is unlikely that I_Ks_ current with relatively fast deactivation kinetics could accumulate during diastole, and as a consequence, it is unlikely that inhibition of current would significantly reduce the reverse use dependence of class III antiarrhythmic agents [7].

These reports have also shown that there are significant species differences in the existence and properties of these two currents. These raise the natural question of how these animal data can be applied to humans. Because it is very difficult to obtain human heart tissue for experimental purposes, especially from healthy donors, the properties of these two important currents in healthy humans have not been known until now. Even the limited data that exist are only from myocardial cells from diseased explanted hearts. 

Beuckelmann et al. wrote that the I_Kr_ component in humans is either absent or very difficult to detect. No signal for the presence of the I_Ks_ component has been detected [21]. Li et al. were the first to demonstrate the presence of both current components in right ventricular myocytes from diseased explanted human hearts [12]. However, in this study, CdCl_2_ and BaCl_2_ were used to inhibit calcium (I_CaL_) and inward rectifying potassium currents (I_K1_), respectively. Specifically, it is known that these agents can significantly alter the kinetic properties of I_Kr_ and I_Ks_ currents. It has been already reported that CdCl_2_ slows down the activation of I_Kr_ currents [23], while BaCl_2_ has a voltage-dependent inhibitory effect on I_K1_, thus interfering with the voltage-dependent study of other currents such as I_Ks_ currents [24]. Therefore, these data should be interpreted with caution. In both studies, the I_Kr_ component is activated with a relatively slow time constant (τ ≈ 200), but the accuracy of these data is questionable due to the presence of a CdCl_2_ blocker in the Ca^2+^ channels. In another study, Veldkamp et al. reported an I_Kr_ activation time constant of 101 ms. In this communication, the presence of an E-4031-insensitive current component (I_Ks_) was also not detected [22].

### 3.2. Comparing Our Results with the Literature

The summary of the results of previous studies on I_Kr_ and I_Ks_ currents highlights an important point: namely, that differences in the cell isolation procedure or in the measurement techniques used can often lead to different and even conflicting results. Therefore, one of our basic controls was that our results obtained on human cardiac myocytes could only be compared with animal results that had been tested using a similar experimental design.

In our studies, the two current components were separated pharmacologically. For this purpose, we used blockers that have been clearly shown in previous studies to have reliable selectivity at the concentrations used [2,11]. In this study, we were able to demonstrate for the first time in a clear and well-detectable manner that both current components, I_Kr_ and I_Ks_, are present in ventricular cells from healthy human hearts. The I_Kr_ current was rapidly activated and slowly deactivated by double-exponential kinetics (Figure 2, left panels). The I_Ks_ current was slowly activated and rapidly deactivated by exponential kinetics (Figure 3, left panels). Previous studies that have detected I_Ks_ current in human hearts have been controversial, as several studies have failed to demonstrate the existence of this current at all [21,22]. However, it should be mentioned that the amplitude of the current recorded in the present study was also very small. However, the control solution in the present study did not contain CdCl_2_ or BaCl_2_, which were included in the study by Li et al. [12]. Thus, the I_Kr_ and I_Ks_ current kinetic parameters reported in this paper are more reliable.

The kinetic parameters of the I_Kr_ and I_Ks_ currents measured in the rabbit and canine ventricular myocytes were highly comparable to those measured in the human ventricular myocytes. In both species, the I_Kr_ component was rapidly activated (τ ≈ 35 ms and 50 ms, respectively) and slowly deactivated in a double-exponential manner (τ_1_ ≈ 650 ms and 800 ms, respectively, and τ_2_ ≈ 6500 ms and 3000 ms, respectively) (Figure 2, middle panels). The I_Ks_ currents were rapidly activated in both the rabbits and dogs (τ ≈ 900 ms and 800 ms, respectively) and deactivated rapidly in an exponential manner (τ ≈ 160 ms and 140 ms, respectively) (Figure 3, middle panels). These canine and rabbit data are also in agreement with those reported in the literature [7,8,10]. Thus, the kinetic properties of the I_Kr_ and I_Ks_ currents measured in the rabbit and canine ventricular myocytes were very similar to those studied in the healthy human ventricular myocytes. The magnitude of the I_Kr_ current was similar in the human and canine myocardial cells, while in the rabbit myocytes, this current was larger (Figure 1, left diagram). The I_Ks_ current was the smallest in the human cells, while in the dogs and rabbits, the magnitudes of the currents were relatively comparable (Figure 1, right diagram).

Comparing our results with the literature, we can say that the I_Kr_ and I_Ks_ amplitudes measured in the rabbits and dogs are in relatively good agreement with those reported by other groups [7,8,10]. However, we found significant differences in the guinea pig experiments. In the guinea pig cardiac myocytes, the I_Kr_ component was activated rapidly but deactivated very slowly with triple-exponential kinetics (Figure 2, right panels, Table 1). The kinetic properties of the I_Ks_ current were also different from those observed in the other three species. The I_Ks_ current was also activated slowly in the guinea pigs, but its kinetics were double-exponential. In contrast, the deactivation of the I_Ks_ current could be best fitted with two exponential functions. It had a fast phase, but it also possessed a late slow phase (Figure 3, right panels, Table 1). These results differ significantly from those reported by Sanguinetti et al., where both activation and deactivation of the I_Kr_ current were fast, whereas activation and deactivation of the I_Ks_ current were slow [11]. These results suggest that I_Ks_ blockers could potentially lengthen the APD in a reverse frequency-independent manner (Sanguinetti hypothesis) [13]. In our measurements, the kinetics of the I_Ks_ component were also slow; however, in contrast to the study reported by Sanguinetti et al. [11], our measurements also showed that the kinetics of I_Kr_ current deactivation were extremely slow. This leads us to conclude that it is possible that the original Sanguinetti hypothesis was based on an incorrect observation. It is true that I_Ks_ current can accumulate during diastole due to slow deactivation, but it is unlikely that the magnitude of this accumulation is large enough to counteract the reverse frequency-dependent APD lengthening caused by I_Kr_ inhibition, which also has slow deactivation. In our experiments, we blocked I_Ks_ current with L-735,821, which has been shown to be the most selective agent to date. It already fully inhibited I_Ks_ at low concentrations of 100 nM [20], thus allowing us to investigate the pure I_Kr_ current.

Finally, it should be noted that the amplitudes of the I_Kr_ and I_Ks_ currents recorded in the guinea pigs were also very different compared to those of the other three species. In the guinea pigs, the amplitudes of both the I_Kr_ and I_Ks_ currents were at least three times higher than those measured in the dogs and humans, but they were also much higher than those observed in the rabbits (Figure 1). In particular, we would like to highlight the magnitude of the I_Ks_ current, as this turns out to be an extremely large current in guinea pigs. In any case, this fact played an important role in the Sanguinetti hypothesis [15]. In contrast, in other species, especially in humans, although undoubtedly present, the I_Ks_ current is much smaller, and its regulatory role in the repolarization of ventricular tissue must therefore be different from what was previously assumed. 

It is known that even a relatively moderate inhibition of I_Kr_ current of about 30–50% results in a strong but reverse frequency-dependent elongation. In the present study, we have also shown that in all four investigated species, the I_Kr_ blocker E-4031 resulted in significant APD lengthening, although to different extents (Figure 4, upper panels). In this study, we presented only representative AP experiments (Figure 4); however, these observations are in good concordance with previous reports where the effects of I_Kr_ and I_Ks_ inhibition on the AP (including cycle length investigations) have been extensively investigated [29,30,31,32]. 

Based on the Sanguinetti hypothesis, it was hypothesized that I_Ks_ inhibitors would also lengthen the APD. Moreover, according to this hypothesis, this elongation would not be reverse frequency-dependent. Verification of this hypothesis was not possible for a long time due to the lack of I_Ks_ inhibitors. However, interestingly, in the present study, the I_Ks_ inhibitor L-735,821 (100 nM) with reliable selectivity did not stretch the APD duration in conventional microelectrode technique experiments on human, dog and rabbit right ventricular papillary muscles (Figure 4, three left panels). This may be explained by the properties of the I_Ks_ current in these species. The current is activated very slowly, and its amplitude is small. Due to its slow activation kinetics, I_Ks_ current is only activated to a very small extent in the voltage and time range relevant for the action potential. Therefore, its inhibition did not result in any measurable APD lengthening [29,30,31]. In another previous study, we showed that when the action potential was artificially stretched, thus allowing the opening of more I_Ks_ channels, the magnitude of the I_Ks_ current was already sufficiently increased to allow for its inhibition to result in significant repolarization elongation [29,30].

Based on these experiments, our group hypothesized that I_Ks_ current plays a minor role in the repolarization of the “normal” healthy heart, but in the case of pathologically long APs (e.g., in the case of drug-induced or genetically prolonged long QT), it may serve as a safety mechanism to control excessive repolarization [29,30,33]. Therefore, the inhibition of this (I_Ks_) current would not result in a significant antiarrhythmic effect. Instead, inhibition of this protective mechanism only leads to a further prolongation of the action potential; i.e., to an increase in proarrhythmic risk [15,29]. Some investigators have suggested that I_Ks_ thus provides a “repolarization reserve” when other outward repolarizing currents are reduced, e.g., by remodeling ion currents during heart failure progression [33,34,35,36].

However, in the present study, we only wanted to point out the physiological observation that in humans, dogs, and rabbits, where the amplitude of the slowly activating I_Ks_ current is not significant, inhibition of the current does not lead to a significant prolongation [7,29,31]. In guinea pigs, however, this current presumably has a different role. Thus, unlike in the other species, the I_Ks_ current magnitude is significantly larger and activates even in the voltage and time range relevant for the action potential [19,37]. Accordingly, selective inhibition of the I_Ks_ current in guinea pigs by the low concentrations (10 µM) of chromanol 293B (at this concentration, chromanol blocks about 60–70% of the I_Ks_ current [37,38]) resulted in a significant (about 12%) APD prolongation (Figure 4, lower right panel), which means that the I_Ks_ current in guinea pigs play an important in normal physiological ventricular action potential repolarization. 

We must emphasize that in a study by Lo et al. [26], the selectivity of chromanol was questioned, where it was reported that chromanol 293B reduced the amplitude of the I_K_ current in H9c2 cells (rat embryonic heart-derived H9c2). However, they did not separate the rapid and slow components of the delayed rectifier currents [26]. In an additional study, we investigated and analyzed the effects of 10 µM chromanol 293B on all other important transmembrane ionic currents (I_Kr_, I_to_ and I_K1_). We have reported that 10 µM chromanol did not affect I_Kr_ and I_to_ currents, and it inhibited I_Ks_ current amplitude by about 60% [38]. Therefore, we can conclude that the observed 12% repolarization lengthening effect induced by I_Ks_ inhibition on the APD in guinea pig ventricular muscle (Figure 4, bottom right panel) might even be slightly underestimated, which demonstrates the importance of the role of I_Ks_ current in guinea pig ventricular AP repolarization.

## 4. Conclusions

Based on our studies, we can conclude that both components of the late rectifier potassium current (I_Kr_ and I_Ks_) are present in the human ventricular myocardium. The I_Kr_ component is activated rapidly and deactivated slowly by double-exponential kinetics, whereas the I_Ks_ component is activated slowly and deactivated rapidly by monoexponential kinetics. When these results are compared with the properties of the I_Kr_ and I_Ks_ currents studied in three animal species widely used in antiarrhythmic drug research, it can be concluded that the I_Kr_ and I_Ks_ currents in human myocardium are similar to those detected in canine and rabbit ventricular myocytes but significantly different from those in guinea pigs.

For pre-clinical testing, several animal species are used. The choice of species markedly influences experimental outcomes and the extrapolation of results to human clinical settings. Based on the result of present study, we argue that dogs and rabbits are useful species for electrophysiological, pharmacological antiarrhythmic, and pro-arrhythmic investigations, but not guinea pigs. 

## 5. Materials and Methods

The experiments were performed on ventricular preparations (papillary myocardial remnants and myocardial cells, respectively) from canine, rabbit, guinea pig, and healthy human donor hearts. Due to the rare availability of human tissue, the time frame of collecting human data was from 1997 until 2015, while the experimental works in animal models were carried out from 2008 to 2020.

### 5.1. Animals

Untreated adult mixed-breed dogs weighing 8–20 kg, domestic New Zealand rabbits weighing 1.5–2 kg, and guinea pigs weighing 300–500 g were used. The protocols were approved by the Review Board of the Department of Animal Health and Food Control of the Ministry of Agriculture and Rural Development, Hungary (XII./01031/000/2008 and XIII./1211/2012 and XIII./3331/2017). The animals were treated with an IV injection of 400 IU/kg heparin before the experiments. Then, the animals sacrificed via cervical dislocation (rabbit or guinea pig) or an overdose of 30 mg/kg sodium pentobarbital (dog), and the hearts were removed via a left thoracotomy and flushed in a physiological nutrient solution at 4 °C.

### 5.2. Healthy Human Heart Muscle Preparations

The investigations conformed to the principles of the Declaration of Helsinki. The experimental protocols were approved by the University of Szeged and National Scientific and Research Ethical Review Boards (Nos. 51-57/1997OEj and 4991-0/2010-1018EKU (339/PI/010)). Proper consent was obtained for use of each individual’s tissue for experimentation.

The myocardial scaffolds for the experiment were obtained from the trimmings of healthy human donor hearts used for aortic valve implantations. Before the hearts were removed, the organ donors did not receive any drug treatments other than furosemide, dobutamine, and plasma. After removal of the valves for the transplantation surgery, the remaining myocardial tissue was stored in cold (4–6 °C) cardioplegic solution until the electrophysiological studies (preparation and myocardial cell isolation) were performed. 

### 5.3. Ion Current Measurement Using the Patch Clamp Technique

Isolated human dog, rabbit, and guinea pig cardiac myocytes were obtained via enzymatic digestion. The cell separation procedure has been described in detail in previous publications [29,30,32,35]. The cells were placed in an organ bath fixed to the stage of a Nikon TMS and Olympus IX51-type inverted microscope and incubated with standard 36–37 °C Tyrode’s solution (NaCl: 135 mM/L, KCl: 4.7 mM/L, KH_2_PO_4_: 1.2 mM/L, MgSO_4_: 1.2 mM/L, HEPES: 10 mM/L, NaHCO_3_: 4.4 mM/L, glucose: 10 mM/L, CaCl_2_:1.8 mM/L; pH 7.2 adjusted with NaOH). A patch clamp micropipette with a resistance of 2.0–2.5 MOhm was filled with the following solution: K-aspartate: 100 mM/L, KCl: 45 mM/L, K2ATP: 3 mM/L, MgCl_2_: 1 mM/L, EGTA: 10 mM/L, HEPES: 5 mM/L (pH: 7.2, adjusted with KOH). In the experiments, the calcium L-type current (I_CaL_) was blocked by adding 1 µM of nisoldipine (Bayer AG, Leverkusen, Germany) to the extracellular solution, and the sodium current (I_Na_) was inactivated by a 20 ms prepulse voltage step to −40 mV. At this voltage, the transient outward potassium current (I_to_) was also largely inactivated. The membrane currents were measured using Axopatch-1D and Axopatch 200B patch clamp amplifiers (Axon Instruments, Union-City, CA, USA) in the whole-cell configuration of the patch clamp technique. Cell capacitance was measured by applying a 10 mV hyperpolarizing pulse from −10 mV. The holding potential was −90 mV. The capacity was measured via the integration of the capacitive transient divided by the amplitude of the voltage step (10 mV). During the measurements, the resistance of 4–8 MOhms was compensated to 50–80%. The results of experiments where the series resistance increased significantly during the measurement were excluded from the average. 

The current measurements were evaluated using the same software (Axon pClamp 7.0–11.0). The experiments were performed at physiological temperature (37 °C). Since the I_Kr_ and I_Ks_ components (in humans and dogs) are relatively small under the depolarizing test pulse, and because other currents (transient outward K^+^, non-specific cation, chloride and Na/Ca exchanger currents) could be also activated during depolarizing pulses and during the time course of experiments, the deactivating tail current observed after the end of the test pulse was measured to assess the I_Kr_ and I_Ks_ currents. In our studies, the two current components were separated pharmacologically. For this purpose, we used blockers that have been clearly shown in previous studies to have reliable selectivities at the concentrations used. Therefore, in our studies, when the I_Kr_ current was measured, the control solution contained the selective I_Ks_ blocker L-738,821 (100 nM, Merck-Sharpe & Dohme, West Point, PA, USA) [20] or chromanol (10–30 µM, Hoechst-Marion-Roussel, Frankfurt, Germany). In contrast, when the I_Ks_ current was measured, the control solution contained the selective I_Kr_ blocker 1–5 µM E-4031 (Institute of Pharmaceutical Research, Budapest, Hungary) [7,11]. This experimental set-up allowed us to record the pure, so-called native I_Kr_ or I_Ks_ currents without the “contaminating” effects of other currents negatively affecting the results. 

### 5.4. Action Potential Measurement Using Conventional Microelectrode Techniques

For the experiments, right ventricular papillary muscle was prepared from canine, rabbit, and guinea pig hearts and healthy human myocardial tissue samples and fixed in an organ bath perfused with Locke solution (NaCl, 115; KCl, 4; CaCl_2_, 1.8; MgCl_2_, 1; NaHCO_3_, 20; glucose, 11) at 37 °C and oxygenated with 95% O_2_ and 5% CO_2_ gas. The preparations were stimulated with square-wave pulses of a 2 ms duration with a double threshold potential using a bipolar platinum electrode (Hugo Sachs Elektronik Stimulator, Type 215/II, March-Hugstetten, Germany). The stimulation cycle length was 1000 ms (1 Hz frequency). The experiments were preceded by a 60 min incubation period. The transmembrane action potentials were recorded using a conventional glass capillary microelectrode with a resistance of 5–20 MOhm, filled with 3 M/l KCl, connected to the input of a high-resistance amplifier (Biologic Amplifier VF 102, Claix, France) and monitored using an oscilloscope (Tetronix 2230).

The electrical signal was fed into an IBM-compatible computer after analog-to-digital conversion (ADA 3300 Data Acquisition Board, Real Time Devices Inc, State College, PA, USA). The maximum diastolic potential, the amplitude of the action potential, and its corresponding duration of 90% repolarization (APD) were measured using an evaluation software (Hugo Sachs Elektronik, Action Potential Evaluation System, version 1.0) developed at the institute. The effects of the tested agents on the action potential and its parameters were measured after a 40 min incubation period.

### 5.5. Statistics

The results are expressed as means ± SEM. The normality of the distributions was verified using the Shapiro–Wilk test, and the homogeneity of the variances was verified using Bartlett’s test in each treatment group. Statistical comparisons were made using an analysis of variance (ANOVA) for repeated measurements followed by Bonferroni’s post hoc test. We controlled for the differences between humans and dogs (* denotes significance), humans and rabbits (* denotes significance), and humans and guinea pigs (* denotes significance). The following symbols were used to denote significant differences: *, ^#^, ^§^ *p* < 0.05; **, ^##^, ^§§^ *p* < 0.01; or when ***, ^###^, ^§§§^ *p* < 0.001.

## Figures and Tables

**Table 1:** Time constants of activation and deactivation kinetics of I_Kr_ and I_Ks_ currents in isolated human, canine, rabbit, and guinea pig ventricular myocytes.

Species	I_Kr_ Current		I_Ks_ Current	
	Activation Time Constant (ms)	Deactivation Time Constant (ms)	Activation Time Constant (ms)	Deactivation Time Constant (ms)
Human	τ = 36.6 ± 3.2 (*n* = 6/3)	τ_1_ = 600.0 ± 53.9 τ_2_ = 6792 ± 876 (*n* = 5/3)	τ = 912.8 ± 198.1 (*n* = 6/4)	τ = 121.9 ± 13.9 (*n* = 7/4)
Dog	τ = 53.8 ± 5.8 (*n* = 15/4)	τ_1_ = 360.3 ± 26.3 τ_2_ = 3310 ± 280 (*n* = 15/4)	τ = 1046 ± 103 (*n* = 8/5)	Τ = 88.6 ± 12.3 (*n* = 8/5)
Rabbit	τ = 35.5 ± 3.1 (*n* = 26/6)	τ_1_ = 641.5 ± 29.0 τ_2_ = 6531 ± 343 (*n* = 35/7)	τ = 888.1 ± 48.2 (*n* = 21/6)	τ = 157.1 ± 4.7 (*n* = 22/6)
Guinea pig	τ = 30.5 ± 2.1 (*n* = 26/3)	τ_1_ = 140.3 ± 10.1 τ_2_ = 800 ± 13 τ_3_ = 6600 ± 600 (*n* = 10/4)	τ_1_ = 500.7 ± 20.1 τ_2_ = 3000 ± 100 (*n* = 10/3)	τ_1_ = 160.6 ± 10.3 τ_2_ = 600.6 ± 100.5 (*n* = 10/3)

The number of values (*n*), reflecting the cell number/animal, is given separately.

## Data Availability

Data is contained within the article.
